# Novel Metabolic Subtypes in Pregnant Women and Risk of Early Childhood Obesity in Offspring

**DOI:** 10.1001/jamanetworkopen.2023.7030

**Published:** 2023-04-04

**Authors:** Ellen C. Francis, Katerina Kechris, Thomas Jansson, Dana Dabelea, Wei Perng

**Affiliations:** 1 The Lifecourse Epidemiology of Adiposity and Diabetes (LEAD) Center, Aurora, Colorado; 2 Department of Biostatistics and Informatics, Colorado School of Public Health, Aurora; 3 Department of Obstetrics and Gynecology, University of Colorado Anschutz Medical Campus, Aurora; 4 Department of Epidemiology, Colorado School of Public Health, University of Colorado Denver Anschutz Medical Campus, Aurora

## Abstract

**Importance:**

The in utero metabolic milieu is associated with offspring adiposity. Standard definitions of maternal obesity (according to prepregnancy body mass index [BMI]) and gestational diabetes (GDM) may not be adequate to capture subtle yet important differences in the intrauterine environment that could be involved in programming.

**Objectives:**

To identify maternal metabolic subgroups during pregnancy and to examine associations of subgroup classification with adiposity traits in their children.

**Design, Setting, and Participants:**

This cohort study included mother-offspring pairs in the Healthy Start prebirth cohort (enrollment: 2010-2014) recruited from University of Colorado Hospital obstetrics clinics in Aurora, Colorado. Follow-up of women and children is ongoing. Data were analyzed from March to December 2022.

**Exposures:**

Metabolic subtypes of pregnant women ascertained by applying k-means clustering on 7 biomarkers and 2 biomarker indices measured at approximately 17 gestational weeks: glucose, insulin, Homeostatic Model Assessment for Insulin Resistance, total cholesterol, high-density lipoprotein cholesterol (HDL-C), triglycerides, free fatty acids (FFA), HDL-C:triglycerides ratio, and tumor necrosis factor α.

**Main Outcomes and Measures:**

Offspring birthweight *z* score and neonatal fat mass percentage (FM%). In childhood at approximately 5 years of age, offspring BMI percentile, FM%, BMI in the 95th percentile or higher, and FM% in the 95th percentile or higher.

**Results:**

A total of 1325 pregnant women (mean [SD] age, 27.8 [6.2 years]; 322 [24.3%] Hispanic, 207 non-Hispanic Black [15.6%], and 713 [53.8%] non-Hispanic White), and 727 offspring with anthropometric data measured in childhood (mean [SD] age 4.81 [0.72] years, 48% female) were included. We identified the following 5 maternal metabolic subgroups: reference (438 participants), high HDL-C (355 participants), dyslipidemic–high triglycerides (182 participants), dyslipidemic–high FFA (234 participants), and insulin resistant (IR)–hyperglycemic (116 participants). Compared with the reference subgroup, women in the IR-hyperglycemic and dyslipidemic–high FFA subgroups had offspring with 4.27% (95% CI, 1.94-6.59) and 1.96% (95% CI, 0.45-3.47) greater FM% during childhood, respectively. There was a higher risk of high FM% among offspring of the IR-hyperglycemic (relative risk, 8.7; 95% CI, 2.7-27.8) and dyslipidemic–high FFA (relative risk, 3.4; 95% CI, 1.0-11.3) subgroups; this risk was of greater magnitude compared with prepregnancy obesity alone, GDM alone, or both conditions.

**Conclusions and Relevance:**

In this cohort study, an unsupervised clustering approach revealed distinct metabolic subgroups of pregnant women. These subgroups exhibited differences in risk of offspring adiposity in early childhood. Such approaches have the potential to refine understanding of the in utero metabolic milieu, with utility for capturing variation in sociocultural, anthropometric, and biochemical risk factors for offspring adiposity.

## Key Points

**Question:**

Can metabolic phenotyping of pregnant women, beyond prepregnancy obesity or gestational diabetes status, improve assessment of adiposity risk in offspring?

**Findings:**

In this cohort study of mother-offspring pairs (1325 women and 727 offspring), an unsupervised clustering of maternal biomarkers measured in blood in midpregnancy yielded 5 subgroups of women whose children had increased fat mass percentage and risk of obesity at approximately 5 years of age. Classification to the dyslipidemic–high free fatty acid and insulin resistant–hyperglycemic subgroups had a greater association with offspring adiposity compared with classifying women on the basis of prepregnancy obesity or gestational diabetes.

**Meaning:**

These findings suggest metabolic phenotyping via conventional metabolic biomarkers measured during pregnancy may improve risk stratification of early childhood obesity.

## Teaser

This cohort study evaluates the association between novel metabolic subtypes in pregnant women and the risk of early childhood obesity in offspring.

## Introduction

Childhood obesity is a global health concern that results in premature morbidity across the life course.^1^ A large literature on the in utero origins of childhood obesity suggests that maternal obesity and hyperglycemia may have a role in programming offspring risk of adiposity starting in early life.^2,3^ Observational studies have found independent associations of maternal glucose and body mass index (BMI; calculated as weight in kilograms divided by height in meters squared) with offspring adiposity and metabolic traits, even below thresholds of gestational diabetes (GDM).^4,5,6,7,8,9,10,11,12,13,14,15^ These data suggest other glycemic, as well as nonglycemic metabolic factors, such as lipids and free fatty acids (FFAs),^16,17,18,19,20,21^ may also play important roles in fetal programming that may operate across the continuum of maternal weight status and glucose metabolism in pregnancy.^18,19^

Several studies have examined the association of individual or classes of metabolic biomarkers such as glucose, insulin, lipoproteins, and FFAs with offspring adiposity.^16,22,23,24,25,26^ However, this approach does not account for potential interrelated effects of biomarkers across classes of compounds, which is a limitation that can be overcome by using data reduction or classification techniques.^24,27^ Studying maternal profiles of metabolic compounds during pregnancy can help to capture the context between biomarkers that both do and do not demonstrate individual statistical associations with offspring adiposity.^27^

In this analysis, we used an unsupervised classification approach that applies algorithms to discover hidden patterns in data to generate metabolic subgroups using 9 biomarkers relevant to metabolic health that were measured during midpregnancy. Next, we tested associations of maternal subgroup classification with offspring adiposity at 2 time points in early life. We hypothesized that the unsupervised classification approach would yield distinct subgroups that are associated with differences in offspring adiposity traits (eg, weight, fat mass, and obesity) measured in the neonatal period and early childhood.

## Methods

All participants provided written informed consent, and the study was approved by the Colorado Multiple Institutional Review Board. The Healthy Start Study is registered as an observational study.^58^ This study followed the Strengthening the Reporting of Observational Studies in Epidemiology (STROBE) reporting guidelines for cohort studies.

### Setting and Participants

The Healthy Start Study is an observational, prebirth cohort that enrolled pregnant women from 2010 to 2014. Inclusion criteria were being aged 15 years or older, no history of stillbirths, being less than 24 weeks’ gestation, singleton birth, and no preexisting serious chronic disease. In-person visits were completed during midpregnancy (median [IQR], 17 [5.4] weeks), late pregnancy (median [IQR], 27 [3.9] weeks), at delivery, and in early childhood (median [IQR] age, 4.6 [0.5] years). A detailed participant flow diagram is included in the eFigure in Supplement 1. Briefly, this analysis included data from 1325 women with assays from blood collected at midpregnancy and offspring anthropometry data collected in the neonatal period (1116 participants) or in childhood (727 participants). The proportion of women who had offspring without neonatal or childhood anthropometric data was not different between the maternal metabolic subgroups (eTable 1 in Supplement 1). Women with offspring who did not have neonatal or childhood anthropometric data were on average younger, with less educational attainment, a lower diet quality, and more likely to have smoked in pregnancy.

### Exposure Derivation: k-Means Clustering and Clustering Inputs

We used unsupervised k-means clustering to identify metabolic subgroups of women. The inputs for k-means clustering comprised 7 biomarkers measured from fasting blood samples collected at approximately 17 gestational weeks, and 2 biomarker indices that have been implicated in in utero metabolic programming. The biomarkers were glucose; total cholesterol (TC); high-density lipoprotein cholesterol (HDL-C); triglycerides (TGs); and FFAs, which were measured using enzymatic kits and the AU400e Chemistry Analyzer (Olympus America Inc); insulin, measured by radioimmunoassay (Millipore); and tumor necrosis factor α (TNF-α), measured using enzyme-linked immunosorbent assay (ELISA) (R&D Systems, Inc). The indices were the ratio of TGs:HDL-C calculated as an indicator of an atherogenic lipid profile^28,29^ and the Homeostatic Model Assessment of Insulin Resistance (HOMA-IR).^30^ We scaled the input variables using min-max scaling to reduce algorithmic selection according to different units of measure and ranges of values.

We then implemented unsupervised k-means clustering on the 9 variables (glucose, insulin, TGs, TC, HDL-C, FFAs, TNF-α, TGs:HDL-C, HOMA-IR) using clusterR.^31^ The optimal number of clusters was determined by the gap statistic which was computed using the factoExtra^32^ algorithm with the following specifications: 25 centroid initializations, 2 to 10 cluster considerations in increments of 1, and 10 Monte Carlo simulated reference data sets. From here forward, we use the term *metabolic subgroup* to refer to the clusters.

### Outcome Measures: Offspring Adiposity

Neonatal body composition, including fat mass (FM) and fat free mass (FFM), was measured by PEA POD (Life Measurement, Inc) air displacement plethysmography. Weight at delivery and gestational age were used to calculate birthweight *z* score and large-for-gestational age status (≥90th percentile) according to national reference data.^33^ In early childhood, FM and FFM were measured using whole body air displacement plethysmography (BodPod, Life Measurement, Inc) with the pediatric option.^34^ The neonatal and childhood fat mass percentage (FM%) was calculated as FM / FFM. Early childhood (mean [SD] age, 4.81 [0.72] years) height was measured to the nearest 0.1 cm by stadiometer, weight was measured to the nearest 0.1 kg using an electronic scale, and age-specific BMI percentiles were calculated according to the US Centers for Disease Control and Prevention reference.^35^

### Covariates: Maternal Demographics and Childhood Obesity Risk Factors

The collection and calculation of maternal perinatal characteristics have been described in detail elsewhere.^22,36,37^ In brief, maternal race and ethnicity, educational attainment, parity, dietary intake, and prenatal smoking status were self-reported via questionnaire. We combined American Indian or Alaska Native, Asian, Native Hawaiian or Pacific Islander, and more than 1 race into a single category of non-Hispanic other. Across all analyses, we consider race and ethnicity as social constructs rather than biological determinants of health.^38^ Race and ethnicity have been associated with prenatal risk, and therefore we included these constructs in the current analysis to assess differences across maternal metabolic subgroup. We calculated maternal prepregnancy BMI using prepregnancy weight and measured height. Gestational weight gain was estimated by subtracting prepregnancy weight from the last clinically measured weight during pregnancy and categorized according to the Institute of Medicine 2009 Guidelines.^39^ All women were screened for GDM at 24 to 28 weeks and GDM diagnosis was abstracted from medical records and according to Carpenter-Coustan criteria.

### Statistical Analysis

First, we examined the distribution of maternal demographic, lifestyle variables, and prenatal risk characteristics across maternal metabolic subgroups to aid in their interpretation. Within each subgroup, we also examined differences in prevalence of women who met criteria for clinically relevant atherogenic dyslipidemia^40,41,42^ and elevated glucose,^40,43,44^ as well as sample-specific cutoffs of the 75th percentile for biomarkers measured at approximately 17 gestational weeks. We assessed statistical significance of differences using a Wald test for continuous variables, and a χ^2^ test for categorical variables.

Next, we examined the association of subgroup membership with continuous neonatal (birthweight *z* score and FM%) and childhood adiposity (BMI and FM%) using linear regression. We estimated the association of subgroup membership and relative risk (RR) of early childhood obesity (BMI ≥95th percentile^35^), and high FM% (FM% ≥95th percentile of the study sample) using linear regression with a Poisson distribution, log link, and repeated subject statement to obtain robust standard errors.^45^ Across all models, we focus on unadjusted estimates given that the maternal subgroups capture metabolic differences related to lifestyle, weight status, and GDM. Accordingly, including these variables as covariates would likely remove meaningful variation from the maternal metabolic subgroups.^46,47^ However, as a sensitivity analysis, we sequentially adjusted multivariable models that account for maternal race and ethnicity, education, parity, smoking, age, prepregnancy BMI, and GDM. To assess whether the association of maternal metabolic subgroup with offspring obesity was predominately explained by maternal obesity or GDM, we excluded women with these conditions in an exploratory analysis.

We performed k-means clustering in R version 4.0.2 (R Project for Statistical Computing). All other statistical analyses were performed with SAS version 9.4 (SAS Institute). We considered a *P* value less than .05 from a 2-sided test to be significant.

## Results

### Participants

The mean (SD) maternal age of participants was 27.8 (6.2) years; 322 (24.3%) were Hispanic, 207 (15.6%) were non-Hispanic Black, 713 (53.8%) were non-Hispanic White; 578 (43.6%) had a college or graduate degree; 53 (4.4%) had diagnosis of GDM; 259 (19.6%) had obesity before pregnancy; and 604 (45.7%) experienced excessive weight gain in pregnancy (Table 1). Approximately half (48.1%) of offspring were female. Mean (SD) birthweight was 3100 (500) grams, and 1.9% were large-for-gestational age. At the early childhood visit the mean (SD) age of offspring was 4.8 (0.7) years, mean (SD) BMI for age percentile was 48.6 (28.6), and 6.2% had obesity.

**Table 1.** Characteristics of 1325 Pregnant Women in the Healthy Start Study, Overall and by Metabolic Subgroup Membership

Maternal characteristics^a^	No. (%)						*P* value
	Full sample (N = 1325)	Reference (n = 438)	High HDL-C (n = 355)	Dyslipidemic–high TG (n = 182)	Dyslipidemic–high FFA (n = 234)	IR-hyperglycemic (n = 116)	
Age, mean (SD), y	27.8 (6.2)	27.7 (6.2)	28.9 (5.9)	28.2 (5.9)	26.5 (6.3)	26.6 (6.2)	<.001
Race and ethnicity							
Hispanic	322 (24.3)	71 (16.2)	66 (18.6)	58 (31.9)	83 (35.5)	44 (37.9)	<.001
Non-Hispanic							
Black	207 (15.6)	85 (19.4)	46 (13.0)	10 (5.5)	41 (17.5)	25 (21.6)	
White	713 (53.8)	257 (58.7)	220 (62.0)	100 (55.0)	100 (42.7)	36 (31.0)	
Other^b^	83 (6.3)	25 (5.7)	23 (6.5)	14 (7.7)	10 (4.3)	11 (9.5)	
Education							
High school or less	428 (32.3)	130 (29.7)	73 (20.6)	71 (39.0)	95 (40.6)	59 (50.9)	<.001
Some college/associate's degree	319 (24.1)	88 (20.1)	87 (24.5)	50 (27.5)	59 (25.2)	35 (30.2)	
College graduate	292 (22.0)	100 (22.8)	91 (25.6)	38 (20.9)	46 (19.7)	17 (14.7)	
Graduate degree	286 (21.6)	120 (27.4)	104 (29.3)	23 (12.6)	34 (14.5)	5 (4.3)	
Nulliparous	636 (48.0)	197 (45.0)	202 (56.9)	74 (40.7)	118 (50.4)	45 (38.8)	<.001
Smoked during pregnancy	121 (9.1)	48 (11.0)	12 (3.4)	27 (14.8)	19 (8.1)	15 (12.9)	<.001
Healthy Eating Index, mean (SD)	54.0 (13.6)	54.4 (14.0)	57.0 (13.4)	53.4 (14.4)	51.5 (12.6)	49.6 (11.8)	<.001
Prepregnancy body mass index^c^							
Mean (SD)	25.7 (6.1)	23.9 (4.4)	23.7 (4.3)	27.3 (5.2)	27.1 (6.7)	33.1 (8.7)	<.001
≥30.0	259 (19.6)	48 (11.0)	26 (7.3)	51 (28.2)	66 (28.3)	68 (59.1)	<.001
Gestational diabetes	53 (4.4)	6 (1.5)	8 (2.4)	12 (7.5)	12 (5.7)	15 (14.6)	<.001
Gestational weight gain^d^							
Insufficient	347 (26.3)	118 (27.0)	81 (22.8)	51 (28.2)	65 (27.9)	32 (27.8)	.11
Adequate	370 (28.0)	133 (30.4)	100 (28.2)	55 (30.4)	62 (26.6)	20 (17.4)	
Excessive	604 (45.7)	186 (42.6)	174 (49.0)	75 (41.4)	106 (45.5)	63 (54.8)	
Biomarkers at approximately 17 gestational weeks^e^							
Glucose ≥95 mg/dL	19 (1.4)	3 (0.7)	1 (0.3)	1 (0.6)	0	14 (12.1)	<.001
Insulin ≥25 uIU/mL	112 (8.5)	1 (0.2)	1 (0.3)	12 (6.6)	3 (1.3)	95 (81.9)	<.001
HOMA-IR ≥2.9	347 (26.2)	54 (12.3)	37 (10.4)	87 (47.8)	53 (22.7)	116 (100.0)	<.001
TGs:HDL-C ≥2.5	366 (27.6)	41 (9.4)	19 (5.4)	181 (99.5)	68 (29.1)	57 (49.1)	<.001
TGs ≥150 mg/dL	320 (24.2)	15 (3.4)	55 (15.5)	168 (92.3)	35 (15.0)	47 (40.5)	<.001
Total-C ≥200 mg/dL	398 (30.0)	10 (2.3)	252 (71.0)	85 (46.7)	31 (13.3)	20 (17.2)	<.001
HDL-C ≤50 mg/dL	284 (21.4)	90 (20.6)	0	81 (44.5)	69 (29.5)	44 (37.9)	<.001
FFAs ≥472 μEq/L	337 (25.4)	1 (0.2)	44 (12.4)	51 (28.0)	197 (84.2)	44 (37.9)	<.001
TNF-α ≥1.36 pg/mL	333 (25.1)	109 (24.9)	83 (23.4)	48 (26.4)	66 (28.2)	27 (23.3)	.71

Abbreviations: FFA, free fatty acid; HDL-C, high-density lipoprotein cholesterol; HOMA-IR, homeostatic model of insulin resistance; IR, insulin resistant; TG, triglyceride; TNF-α, tumor necrosis factor-α; total-C, total cholesterol.

^a^ To convert glucose to millimoles per liter, multiply by 0.0555; to convert insulin to picomoles per liter, multiply by 6.945; to convert TGs to millimoles per liter, multiply by 0.0113; to convert HDL-C to millimoles per liter, multiply by 0.0259.

^b^ Non-Hispanic other: due to low cell sizes for some of the categories, American Indian or Alaska Native, Asian, Native Hawaiian or Pacific Islander, and more than 1 race were combined into a single category of “non-Hispanic other.”

^c^ BMI is calculated as weight in kilograms divided by height in meters squared.

^d^ Gestational weight gain categories classified according to the Institute of Medicine 2009 guidelines.^39^

^e^ Sources of biomarker cutoffs were Grundy et al^40^ for glucose, TGs, and HDL-C; Melmed et al^41^ for insulin and total-C; Salazar et al^42^ for TGs:HDL-C; and Ghasemi et al^43^ and Lee et al^44^ for HOMA-IR.

### Maternal Metabolic Subgroups

The gap statistic indicated an optimal number of 5 metabolic subgroups. We compared the percentage of women in each subgroup that met clinically relevant cutoffs for atherogenic dyslipidemia, elevated glucose, and sample-specific cutoffs to inform the naming of the subgroups. The subgroups were named as follows. The reference group (33% of the sample) included women who had biomarker levels indicative of a favorable metabolic profile. In addition, this subgroup was used as the reference because it was the largest, which provides more stable estimates in pairwise comparisons. The high HDL-C subgroup (27%) contained women who all had HDL-C levels considered to be anti-atherosclerotic and endothelial-protective (HDL-C >50 mg/dL [to convert to millimoles per liter, multiply by 0.0259]).^40^ The dyslipidemic–high TG (14%) was a subgroup of women of whom most had TGs:HDL-C ratio values considered to be atherogenic (TGs:HDL-C ≥2.5).^42^ The dyslipidemic–high FFA (18%) was a subgroup of women of whom most had FFAs in the 75th percentile or higher (≥472 μEq/L). The insulin resistant (IR)–hyperglycemic group (9%) included women who all had HOMA-IR values indicative of insulin resistance (HOMA-IR ≥2.9)^43,44^ (Figure).

**Figure.**
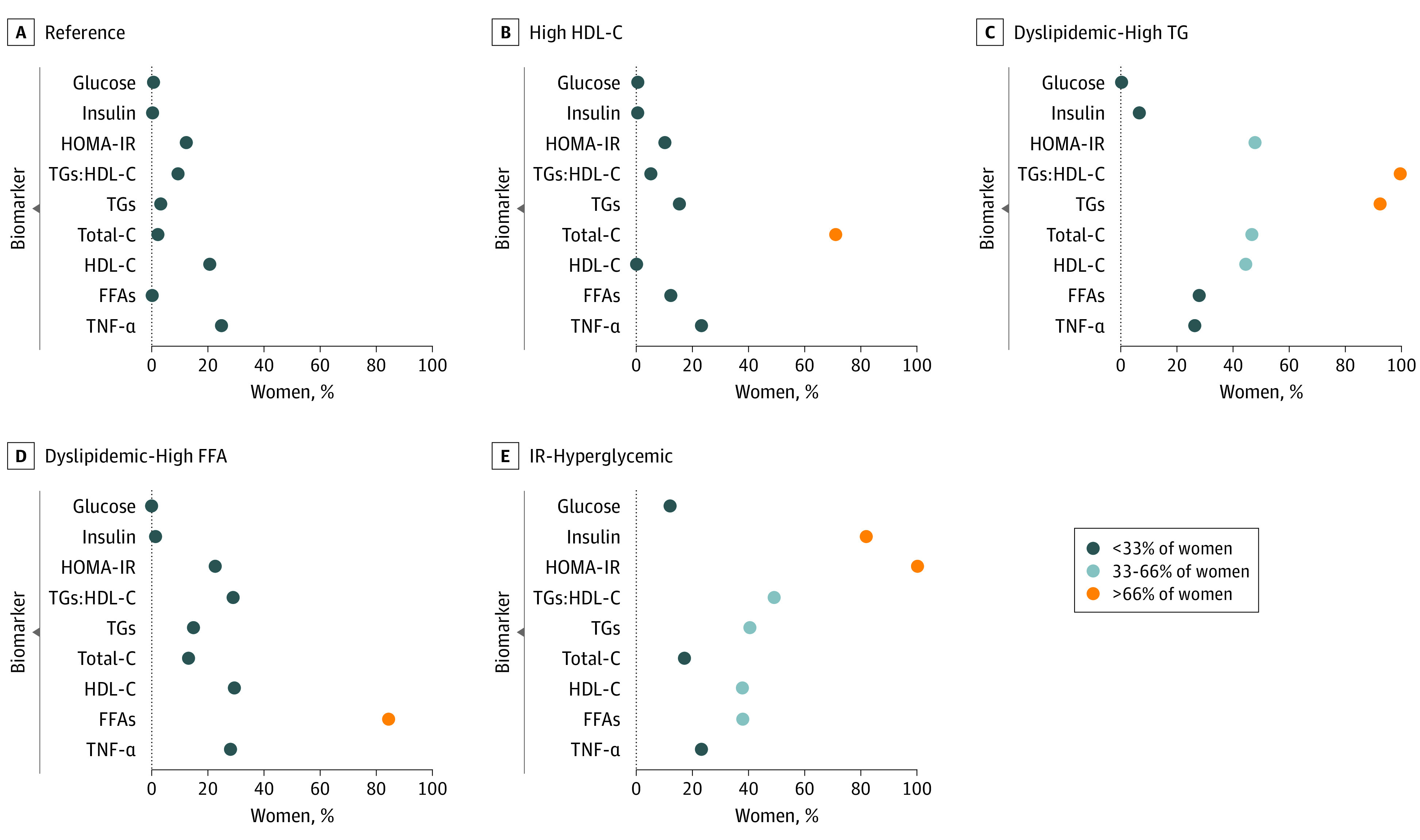
Prevalence of Women Who Met Criteria for Atherogenic Dyslipidemia, Elevated Glucose, and High Sample-Specific Cutoffs for Biomarkers at Approximately 17 Gestational Weeks Dark blue circle: less than 33% of women, light blue: 33% to 66% of women, orange: more than 66% of women. Biomarker cutoffs: glucose greater than or equal to 95 mg/dL (to convert to millimoles per liter, multiply by 0.0555), insulin greater than or equal to 25 uIU/mL (to convert to picomoles per liter, multiply by 6.945), HOMA-IR greater than or equal to 2.9^42,43^, TGs:HDL-C greater than or equal to 2.5^39,40,41^, TGs greater than or equal to 150 mg/dL (to convert to millimoles per liter, multiply by 0.0113), total-C greater than or equal to 200 mg/dL (to convert to millimoles per liter, multiply by 0.0259)^39^, HDL-C less than or equal to 50 mg/dL (to convert to millimoles per liter, multiply by 0.0259)^41^, FFAs greater than or equal to 472 μEq/L, TNF-α greater than or equal to 1.36 pg/mL. FFA indicates free fatty acid; HOMA-IR, homeostatic model of insulin resistance, HDL-C, high-density lipoprotein cholesterol; IR, insulin resistant; TG, triglyceride; TNF-α, tumor necrosis factor-α; total-C, total cholesterol.

Among women in the reference subgroup, there was a low frequency of GDM (6 participants [1.5%]) and a balanced distribution of education status. Women classified to the high HDL-C subgroup had a higher quality diet (Healthy Eating Index score ≥57) and had the lowest prevalence of prepregnancy obesity (26 participants [7.3%]). Almost half of the women classified to the dyslipidemic–high TG group had high HOMA-IR (≥2.9). These women had the highest rates of prenatal smoking (27 participants [14.8%]), lower educational attainment (71 participants [39.0%] had a high school diploma or less), and older age than other subgroups. Women in the dyslipidemic–high FFA had FFAs levels in midpregnancy that were 79.2% greater than the levels of the reference subgroup in late pregnancy (eTable 2 in Supplement 1); however, the remaining biomarker levels of the dyslipidemic–high FFA subgroup were below clinical thresholds of metabolic risk. The women in this subgroup had a mean prepregnancy BMI of 27.1, and 106 partcipants (45.5%) had excessive gestational weight gain. Finally, more than a third of women in the IR-hyperglycemic subgroup had atherogenic lipid levels (TGs ≥150 mg/dL [to convert to millimoles per liter, multiply by 0.0113] and HDL-C ≤50 mg/dL),^40^ 63 participants (54.8%) had excessive gestational weight gain, and 68 participants (59.1%) had prepregnancy obesity. The women in this subgroup had the highest rates of GDM (15 participants [14.6%]), the lowest quality diet (mean Healthy Eating Index score of 50), and the lowest prevalence of nulliparity (45 participants [38.8%]).

### Associations of Maternal Metabolic Subgroups With Offspring Adiposity

Compared with the reference subgroup, offspring of the IR-hyperglycemic subgroup had higher neonatal (β, 1.34; 95% CI, 0.37-2.31) and childhood (β, 4.27; 95% CI, 1.94-6.59) FM%, and higher childhood BMI percentile (β, 16.79; 95% CI, 8.38-25.20) (Table 2). Offspring born to women in the dyslipidemic–high FFA subgroup had higher childhood FM% (β, 1.96; 95% CI, 0.45-3.47). Offspring of the dyslipidemic–high TGs had higher FM% and BMI in childhood; however, the confidence intervals crossed the null for the estimate of BMI. There was no difference in offspring outcomes between the reference and high HDL-C subgroup.

**Table 2.** Association of Maternal Metabolic Subgroup Membership With Neonatal and Offspring Adiposity During Early Childhood (Median Age 5 Years) Among Mother-Offspring Dyads

Offspring outcome, subgroup vs reference	Unadjusted	Adjusted^a^		
		Maternal demographics and lifestyle	Prepregnancy BMI^b^	GDM^c^
Neonatal				
Fat mass, %				
Reference	0 [Reference]	0 [Reference]	0 [Reference]	0 [Reference]
High HDL-C	−0.36 (−0.98 to 0.26)	−0.39 (−1.00 to 0.23)	−0.38 (−0.99 to 0.23)	−0.38 (−1.01 to 0.25)
Dyslipidemic–high TG	0.03 (−0.75 to 0.81)	−0.22 (−1.00 to 0.57)	−0.39 (−1.17 to 0.39)	−0.29 (−1.12 to 0.54)
Dyslipidemic–high FFA	−0.39 (−1.02 to 0.25)	−0.49 (−1.14 to 0.15)	−0.69 (−1.36 to −0.03)	−0.43 (−1.10 to 0.24)
IR-hyperglycemic	1.34 (0.37 to 2.31)	1.16 (0.16 to 2.17)	0.60 (−0.42 to 1.61)	1.02 (−0.02 to 2.07)
Birthweight, *z *score				
Reference	0 [Reference]	0 [Reference]	0 [Reference]	0 [Reference]
High HDL-C	−0.02 (−0.15 to 0.12)	−0.06 (−0.19 to 0.07)	−0.06 (−0.19 to 0.07)	−0.04 (−0.17 to 0.10)
Dyslipidemic–high TG	−0.01 (−0.18 to 0.15)	−0.10 (−0.27 to 0.06)	−0.16 (−0.32 to 0.01)	−0.06 (−0.23 to 0.11)
Dyslipidemic–high FFA	−0.14 (−0.29 to 0.01)	−0.18 (−0.32 to −0.03)	−0.23 (−0.38 to −0.09)	−0.15 (−0.30 to 0.00)
IR-hyperglycemic	0.15 (−0.07 to 0.38)	0.12 (−0.10 to 0.33)	−0.04 (−0.26 to 0.18)	0.13 (−0.09 to 0.35)
Childhood				
Fat mass, %				
Reference	0 [Reference]	0 [Reference]	0 [Reference]	0 [Reference]
High HDL-C	0.53 (−0.74 to 1.80)	0.52 (−0.75 to 1.78)	0.50 (−0.77 to 1.76)	0.44 (−0.86 to 1.73)
Dyslipidemic–high TG	1.70 (0.15 to 3.25)	1.22 (−0.38 to 2.81)	0.96 (−0.66 to 2.58)	1.29 (−0.37 to 2.95)
Dyslipidemic–high FFA	1.96 (0.45 to 3.47)	2.07 (0.62 to 3.53)	1.78 (0.27 to 3.28)	2.27 (0.81 to 3.73)
IR-hyperglycemic	4.27 (1.94 to 6.59)	4.30 (2.03 to 6.56)	3.40 (1.15 to 5.64)	4.18 (1.80 to 6.55)
BMI, percentile^d^				
Reference	0 [Reference]	0 [Reference]	0 [Reference]	0 [Reference
High HDL-C	1.27 (−3.83 to 6.37)	1.76 (−3.30 to 6.82)	1.60 (−3.41 to 6.60)	1.93 (−3.28 to 7.13)
Dyslipidemic–high TG	3.96 (−2.65 to 10.58)	0.88 (−5.52 to 7.27)	−2.41 (−8.81 to 3.99)	2.12 (−4.45 to 8.69)
Dyslipidemic–high FFA	3.72 (−2.54 to 9.98)	1.47 (−4.65 to 7.59)	−1.87 (−8.10 to 4.37)	2.00 (−4.30 to 8.29)
IR-hyperglycemic	16.79 (8.38 to 25.20)	13.22 (4.64 to 21.79)	3.00 (−5.89 to 11.89)	12.54 (3.59 to 21.49)

Abbreviations: BMI, body mass index; FFA, free fatty acid; GDM, gestational diabetes; HDL-C, high-density lipoprotein cholesterol; IR, insulin resistant; TG, triglyceride.

^a^ Maternal demographics and lifestyle model adjusted for maternal race and ethnicity, education, parity, smoking, and age.

^b^ Prepregnancy BMI included the maternal demographics and lifestyle model plus prepregnancy BMI.

^c^ GDM included the maternal demographics and lifestyle model plus GDM.

^d^ BMI is calculated as weight in kilograms divided by height in meters squared; percentiles in childhood according to the US Centers for Disease Control and Prevention reference.^35^

### Comparison of Metabolic Subgroup Membership With Prepregnancy Obesity or GDM in Association With Childhood Adiposity

Compared with offspring of women in the reference subgroup, those born to women in the IR-hyperglycemic subgroup had a nearly 5-fold higher risk of childhood obesity (RR, 5.3; 95% CI, 2.4-11.4) and 9-fold higher risk of high FM% (≥95th percentile) (RR, 8.7; 95% CI, 2.7-27.8). Additionally, children of women in the dyslipidemic–high FFA subgroup had over 3-fold the risk of high FM% (RR, 3.4; 95% CI, 1.0-11.3) (Table 3). The magnitude of RR of childhood obesity with respect to maternal metabolic subgroup membership was larger than that of prepregnancy obesity alone (RR, 2.1; 95% CI, 1.1-4.1), GDM alone (RR, 3.0; 95% CI, 0.9-9.2), or prepregnancy obesity and GDM (RR, 4.0; 95% CI, 1.1-14.8). After excluding women with either prepregnancy obesity or GDM (312 participants), membership to the IR-hyperglycemic subgroup vs reference subgroup remained significantly associated with risk of childhood obesity (RR, 4.9; 95% CI, 1.3-17.9). Although the estimate was somewhat attenuated, it was still greater than the estimate attributable to prepregnancy obesity, GDM, or both conditions. All estimates, regardless of whether the exposure was maternal metabolic subgroup, obesity, or GDM were somewhat attenuated after adjustment for sociodemographic and behavioral prenatal factors associated with risk; however, the association of the IR-hyperglycemic subgroup with offspring obesity remained significant.

**Table 3.** Associations of Maternal Metabolic Subgroup, Prepregnancy Obesity, and Gestational Diabetes (GDM) With Offspring Obesity and High Fat Mass Percentage Among Mother-Offspring Dyads

In utero exposure	Childhood obesity, BMI ≥95th percentile, RR (95% CI)		Childhood fat mass % ≥95th percentile, unadjusted RR (95% CI)
	Unadjusted	Adjusted^a^	
Subgroup vs reference			
Reference	1 [Reference]	1 [Reference]	1 [Reference]
High HDL-C	1.0 (0.4-2.5)	1.1 (0.4-2.8)	2.6 (0.8-8.3)
Dyslipidemic–high TG	1.3 (0.4-3.6)	0.9 (0.3-2.4)	1.3 (0.2-6.9)
Dyslipidemic–high FFA	1.7 (0.7-4.0)	1.2 (0.5-2.7)	3.4 (1.0-11.3)
IR-hyperglycemic	5.3 (2.4-11.4)	3.4 (1.6-7.4)	8.7 (2.7-27.8)
Maternal pregnancy complications vs none			
Neither condition	1 [Reference]	1 [Reference]	1 [Reference]
Prepregnancy obesity only	2.1 (1.1-4.1)	1.6 (0.8-3.2)	3.0 (1.4-6.5)
GDM only	3.0 (0.9-9.2)	2.7 (0.8-8.9)	NA^b^
Obesity and GDM	4.0 (1.1-14.8)	3.3 (0.7-14.3)	3.5 (0.5-23.9)

Abbreviations: BMI, body mass index; FFA, free fatty acid; HDL-C, high density lipoprotein cholesterol; IR, insulin resistant; NA, not applicable; TG, triglyceride.

^a^ Adjusted model includes maternal race and ethnicity, education, parity, smoking, and age.

^b^ Estimate not available because none of the women with GDM had offspring with fat mass percentages greater than or equal to the 95th percentile.

## Discussion

Using prospective data from a prebirth cohort with follow-up through approximately 5 years of age, we identified 5 distinct maternal metabolic subgroups according to 7 biomarkers derived from fasting blood at approximately 17 gestational weeks and 2 biomarker indices. According to the levels of metabolic biomarkers and the prevalence of meeting clinically relevant thresholds for specific biomarkers, we refer to the subgroups as reference, high HDL-C, dyslipidemic–high TGs, dyslipidemic–high FFA, and IR-hyperglycemic. These subgroups exhibited differences in demographic, lifestyle, and prenatal risk factors for offspring obesity (eg, women with lower educational attainment, diet quality, and excessive gestational weight gain). Maternal membership to the IR-hyperglycemic subgroup was associated with the greatest risk of offspring adiposity in the neonatal and childhood periods, and membership to the dyslipidemic–high FFA or IR-hyperglycemic subgroups had a greater association with offspring FM% in the 95th percentile or higher than prepregnancy obesity alone, GDM alone, or prepregnancy obesity and GDM. These data suggest that nuances of maternal metabolism resulting from effects of interrelated biomarkers and across classes of compounds, which are not fully captured by traditional classifications of obese BMI or GDM, play a role in the in utero origins of excess offspring adiposity. In addition, women in these metabolic subgroups display different sociodemographic and lifestyle characteristics that may also be associated with an increased risk of childhood adiposity. In the following sections, we summarize sociodemographic and metabolic characteristics of each subgroup and whether or how they are associated with offspring adiposity.

### Reference Subgroup

Of the 5 subgroups identified by the cluster-based algorithm, we identified a group that comprised the largest proportion of women in the study sample (33%), the majority of whom had biomarker levels below clinical thresholds for metabolic risk,^40,41,42,43,44^ mean prepregnancy BMI within a healthy clinical range (18.5 to 24.9), and the lowest rates of GDM diagnosis across the subgroups. Accordingly, we labeled this group as the reference category for comparisons with other subgroups.

### IR-Hyperglycemic Subgroup

The IR-hyperglycemic subgroup, which comprised 9% of women, was characterized by insulin resistance and an atherogenic lipid profile (high TGs, high FFAs, low HDL-C) in midpregnancy. Classification to this subgroup was associated with higher neonatal and childhood FM%. This finding aligns with prior studies^4,6,8,10,14,22,23,36^ documenting the continuous relationship between maternal glucose levels and insulin resistance in pregnancy with fetal growth and fat mass accretion, including prior studies from our cohort. Furthermore, prior studies^18,19,48,49,50^ have shown that in the context of maternal GDM and obesity, TGs are important drivers of newborn weight^48^ and, at times, may even exhibit greater associations with newborn weight and adiposity than glucose.

### Dyslipidemic–High TGs Subgroup

In our study, offspring of women in the dyslipidemic–high TGs subgroup, comprising 14% of women, had higher FM% and BMI in childhood, although the confidence intervals crossed the null. The association between exposure to high TGs in utero and greater offspring adiposity is consistent with prior data from women with GDM and obesity,^18,19,48,49,50^ and data from 2 general risk pregnancy cohorts measuring TGs in early or midpregnancy.^16,17^ In a subset of women from the Hyperglycemia and Adverse Pregnancy Outcome (HAPO) study, TGs measured at approximately 28 gestational weeks were not associated with BMI *z* score or sum of skinfolds in offspring at age 6 years.^51^ This discrepancy may be due to differences in TGs levels between studies, as the women in the dyslipidemic–high TGs subgroup had TGs that were 30% higher than women in the HAPO subset at a comparable gestational time point. Additionally, TGs in the presence of insulin resistance (see IR-hyperglycemic subgroup) increased risk of offspring adiposity; however, in the absence of higher glycemia, the extent of risk of offspring adiposity was diminished, underscoring a potential synergistic effect of metabolic compounds.

### Dyslipidemic–High FFA Subgroup

The dyslipidemic–high FFA subgroup, which comprised 18% of the sample, generally had favorable levels of metabolic markers except for strikingly high levels of FFAs. In this subgroup, the FFA levels in midpregnancy were already higher than the FFA levels of women in the reference subgroup in late pregnancy, which is noteworthy considering the increase in lipolysis and circulating FFAs as gestation progresses.^52^ Unlike the IR-hyperglycemic subgroup, classification to the dyslipidemic–high FFA subgroup was not associated with differences in FM% in the neonatal period. However, in early childhood, offspring of the dyslipidemic–high FFA subgroup had higher FM% and higher risk for FM in the 95th percentile or higher. In our cohort, prior analyses have shown that associations of maternal lipoproteins and FFAs with offspring metabolic traits are apparent in childhood^53^ rather than with offspring adiposity at birth.^22,36^ Studies that assessed FFAs in pregnancy in association with newborn body composition have yielded conflicting results.^23,26,54^ However, prior studies have found similar positive associations of FFAs, as well as n-6 polyunsaturated fatty acid patterns and individual n-6 polyunsaturated fatty acids during pregnancy with offspring body composition when measured in early childhood.^16,20,21,24^ The differences in findings across studies demonstrate the importance of longitudinal birth cohorts and measurement of offspring adiposity at multiple time points across early life, as not all manifestations of in utero exposures are apparent at birth.

### High HDL-C Subgroup

Finally, we identified a high HDL-C subgroup, which included 27% of women in the sample. The majority of women in this subgroup had biomarker levels below clinical thresholds for metabolic risk,^40,41,42,43,44^ mean prepregnancy BMI within the normal weight range, and lower rates of GDM diagnosis. Offspring of women in this group exhibited no difference in adiposity at birth or in early childhood from that of the reference group. The lack of differences could be due to the similarity in metabolic profiles between the women in the reference and high HDL-C subgroups, and highlights the importance of maternal metabolic health for in utero programming.

### Comparison of Subgroup Membership with Traditional Prenatal Risk Factors for Childhood Obesity

Our findings support growing evidence for the importance of identifying associated compounds and interrelated biomarkers to capture meaningful metabolic heterogeneity within a population. Furthermore, differences in traditional metabolic biomarkers across the subgroups indicate the involvement of both glycemic and nonglycemic metabolic factors to fetal programming of adipogenic potential that operates across the continuum of maternal weight status and glucose metabolism in pregnancy.^55^

Additionally, the association of maternal subgroups with childhood obesity remained significant, even following adjustment for known sociodemographic and behavioral prenatal risk factors, including race and ethnicity, education, and prenatal smoking. On the other hand, neither maternal obesity nor GDM were associated with offspring obesity after adjustment for the same sociodemographic and lifestyle variables. These findings suggest that the subgroups characterized herein have utility for identifying pregnant women whose offspring are at elevated risk of early-life adiposity, above and beyond traditional risk factors. Together, our findings indicate that unsupervised clustering approaches of biomarkers in pregnancy have potential to capture the relative risk or relative contribution of in utero metabolic differences in pregnancy to offspring adiposity. Applications of these approaches are needed in future studies to assess whether this residual variation in the standard categories of risk is relevant for clinicians to consider in future efforts to refine risk classification in pregnancy.

### Limitations

This study had limitations. We did not have childhood anthropometric data on all offspring of women included in this analysis. Women whose offspring did not have data on the adiposity outcomes of interest had a lower diet quality, educational attainment, and reported smoking in pregnancy. These characteristics have been associated with offspring obesity, and therefore the estimates in the current study may underrepresent the magnitude of risk attributable to the in utero environment. A limitation of the k-means clustering algorithm is the need to predefine a value for *k*, and thus classification of individuals can change according to the specified *k*,^56^ which may challenge replicability of subgroups across studies. Although there is no standard for validation of clustering classification, we used the gap statistic which captures cluster cohesion and distinctness to inform the value for *k*.^56^ In addition, we overlaid data on lifestyle and prenatal risk to capture some of the complexities between individual behaviors and metabolic compounds, which adds value to automated classification methods.^57^

## Conclusions

The data from the current study suggest that unsupervised clustering methods may be a useful technique for identifying co-occurring patterns of metabolic markers measured during pregnancy that capture variation in sociocultural, anthropometric, and biochemical risk factors for excess adiposity in offspring. However, our findings need to be confirmed in a separate cohort. Moreover, given that we studied a group of fairly healthy pregnant women, additional research is warranted to assess validity of these subgroups in other populations with more complex obstetric presentation, and replicability of their associations with offspring adiposity.

## Notes

Supplement 1. **eTable 1. **Characteristics of 1,325 Pregnant Women in the Healthy Start Study by Availability of Offspring Anthropometry Data at the Early Childhood Visit **eTable 2. **Mean ± SD of Metabolic Markers Among 1,325 Pregnant Women in the Healthy Start Study, Overall and According to Metabolic Subgroup Membership **eFigure. **Flow Diagram of the Analytical Sample of Pregnant Women and Their Offspring in the Healthy Start Study

Supplement 2.
Data Sharing Statement
