# Supplementary material for: Novel Metabolic Subtypes in Pregnant Women and Risk of Early Childhood Obesity in Offspring
Source: JAMA Netw Open. 2023 Apr 4;6(4):e237030. doi: 10.1001/jamanetworkopen.2023.7030 (PMC10074224; doi:10.1001/jamanetworkopen.2023.7030)
Supplement: Supplement 1. — eTable 1. Characteristics of 1,325 Pregnant Women in the Healthy Start Study by Availability of Offspring Anthropometry Data at the Early Childhood Visit eTable 2. Mean ± SD of Metabolic Markers Among 1,325 Pregnant Women in the Healthy Start Study, Overall and According to Metabolic Subgroup Membership eFigure. Flow Diagram of the Analytical Sample of Pregnant Women and Their Offspring in the Healthy Start Study [file jamanetwopen-e237030-s001.pdf]

Supplemental Online Content

Francis EC, Kechris K, Jansson T, Dabelea D, Perng W. Novel metabolic subtypes in pregnant women and risk of early childhood obesity in offspring. *JAMA Netw Open*. 2023;6(4):e237030. doi:10.1001/jamanetworkopen.2023.7030

eTable 1. Characteristics of 1,325 Pregnant Women in the Healthy Start Study by Availability of Offspring Anthropometry Data at the Early Childhood Visit

eTable 2. Mean ± SD of Metabolic Markers Among 1,325 Pregnant Women in the Healthy Start Study, Overall and According to Metabolic Subgroup Membership

eFigure. Flow Diagram of the Analytical Sample of Pregnant Women and Their Offspring in the Healthy Start Study

This supplemental material has been provided by the authors to give readers additional information about their work.

© 2023 Francis EC et al. *JAMA Network Open.*

eTable 1. Characteristics of 1,325 Pregnant Women in the Healthy Start Study by Availability of Offspring Anthropometry Data at the Early Childhood Visit

Maternal Characteristics:	Early childhood anthropometric data		P-value
	Yes (N=727)	No (N=598)	
Maternal subgroup			0.94
Insulin sensitive (Reference)	33.4 (243)	32.6 (195)	
High HDL-C	13.3 (97)	14.2 (85)	
Dyslipidemic-High TG	27.1 (197)	26.4 (158)	
Dyslipidemic-High FFA	8.3 (60)	9.4 (56)	
IR-Hyperglycemic	17.9 (130)	17.4 (104)	
Age, years; mean ± SD	28.3 ± 6.1	27.1 ± 6.2	<0.001
Race/ethnicity (%, n)			0.22
Hispanic	23.8 (173)	24.9 (149)	
Non-Hispanic Black	15.1 (110)	16.2 (97)	
Non-Hispanic White	55.6 (406)	51.3 (307)	
Non-Hispanic other	5.2 (38)	7.5 (45)	
Education (%, n)			0.01
High school or less	28.8 (209)	36.6 (219)	
Some college/assoc. degree	24.2 (176)	23.9 (143)	
College graduate	21.9 (159)	22.2 (133)	
Graduate degree	25.1 (183)	17.2 (103)	
Nulliparous (%, n)	48.8 (355)	45.3 (281)	0.50
Smoked during pregnancy (%, n)	6.5 (47)	12.4 (74)	<0.001
Healthy Eating Index; mean ± SD	54.8 ± 13.4	53.1 ± 13.9	0.03
Prepregnancy BMI ≥30.0 kg/m^2 (%, n)	20.1 (146)	19.0 (113)	0.10
Gestational Diabetes Mellitus (%, n)	4.8 (33)	3.7 (19)	0.35

Non-Hispanic other: due to low cell sizes for some of the categories, we combined American Indian or Alaska Native, Asian, Native Hawaiian or Pacific Islander and >1 race into a single category of “non-Hispanic other.” P-value from Chi-square tests for categorical variables and Type3 tests for significant main effects for continuous variables.

© 2023 Francis EC et al. *JAMA Network Open.*

eTable 2. Mean ± SD of Metabolic Markers Among 1,325 Pregnant Women in the Healthy Start Study, Overall and According to Metabolic Subgroup Membership

Maternal metabolic marker:	Full Sample	Reference	High HDL-C	Dyslipidemic-High TG	Dyslipidemic-High FFA	Insulin Resistant-Hyperglycemic	P
**Median 17 gestational weeks**							
Glucose, mg/dL	76.7 ± 6.9	76.2 ± 6.3	75.0 ± 5.8	77.8 ± 6.3	75.0 ± 6.2	85.6 ± 7.9	<0.001
Insulin, uIU/mL	13.2 ± 8.3	10.3 ± 4.0	10.2 ± 4.1	15.4 ± 5.7	11.7 ± 5.0	32.7 ± 11.0	<0.001
HOMA-IR	2.5 ± 1.8	1.9 ± 0.8	1.9 ± 0.8	3.0 ± 1.2	2.2 ± 0.9	6.9 ± 2.3	<0.001
TGs:HDL-C	2.1 ± 1.1	1.7 ± 0.6	1.6 ± 0.5	4.0 ± 1.3	2.1 ± 0.7	2.7 ± 1.1	<0.001
TGs, mg/dL	122.7 ± 49.6	94.1 ± 26.6	115.2 ± 32.0	202.6 ± 54.4	115.9 ± 30.9	142.6 ± 44.9	<0.001
Total-C, mg/dL	182.1 ± 35.8	159.0 ± 22.1	214.7 ± 26.9	196.2 ± 36.2	170.3 ± 27.2	171.8 ± 32.7	<0.001
HDL-C, mg/dL	61.1 ± 12.9	58.1 ± 9.2	74.6 ± 10.2	52.6 ± 9.4	55.9 ± 9.9	55.2 ± 11.0	<0.001
FFAs, uEq/L	375.8 ± 167.9	266.1 ± 94.4	341.6 ± 119.3	405.1 ± 138.3	590.1 ± 134.4	416.9 ± 185.0	<0.001
TNF-α	1.3 ± 1.3	1.3 ± 1.3	1.2 ± 1.1	1.4 ± 1.8	1.4 ± 1.5	1.3 ± 1.1	0.59
**Median 27 gestational weeks**							
Glucose, mg/dL	78.0 ± 8.5	76.7 ± 7.4	76.5 ± 7.1	81.2 ± 9.0	76.9 ± 6.6	85.4 ± 12.9	<0.001
Insulin, uIU/mL	17.9 ± 16.8	15.2 ± 19.0	14.1 ± 11.5	22.9 ± 18.5	17.5 ± 9.7	33.7 ± 20.1	<0.001
HOMA-IR	3.6 ± 4.4	3.0 ± 5.0	2.8 ± 2.8	4.8 ± 5.0	3.4 ± 2.1	7.4 ± 5.9	<0.001
HbA1C, %	2.8 ± 1.4	2.3 ± 1.0	2.2 ± 0.9	4.6 ± 1.8	2.8 ± 1.1	3.3 ± 1.5	<0.001
TGs:HDL-C	5.0 ± 0.3	4.9 ± 0.3	5.0 ± 0.3	5.0 ± 0.3	5.0 ± 0.3	5.2 ± 0.4	<0.001
TGs, mg/dL	163.2 ± 62.0	137.5 ± 42.7	154.7 ± 50.5	241.4 ± 70.5	157.2 ± 49.7	177.0 ± 63.9	<0.001
Total-C, mg/dL	211.3 ± 40.9	195.0 ± 31.0	241.0 ± 36.4	219.3 ± 41.8	198.3 ± 34.8	189.8 ± 39.6	<0.001
HDL-C, mg/dL	63.4 ± 13.4	61.9 ± 10.9	73.9 ± 12.0	54.6 ± 10.7	59.8 ± 12.2	56.8 ± 11.8	<0.001
FFAs, uEq/L	372.4 ± 147.1	331.1 ± 136.3	381.0 ± 145.5	405.5 ± 148.3	405.1 ± 156.8	385.6 ± 135.9	<0.001
TNF-α, pg/mL	1.4 ± 1.5	1.5 ± 1.5	1.4 ± 1.2	1.4 ± 2.1	1.4 ± 1.2	1.3 ± 1.1	0.86

Abbreviations: FFAs, free fatty acids; HbA1C, hemoglobin A1C; HDL-C, high density lipoprotein cholesterol; HOMA-IR, homeostatic model of assessment-insulin resistance; Total-C, total cholesterol; TGs, triglycerides; TNFα, tumor necrosis factor-α

Pvalue represents a test for significant main effects of the maternal metabolic subgroup (Type3)

© 2023 Francis EC et al. *JAMA Network Open.*

eFigure 1. Flow Diagram of the Analytical Sample of Pregnant Women and Their Offspring in the Healthy Start Study

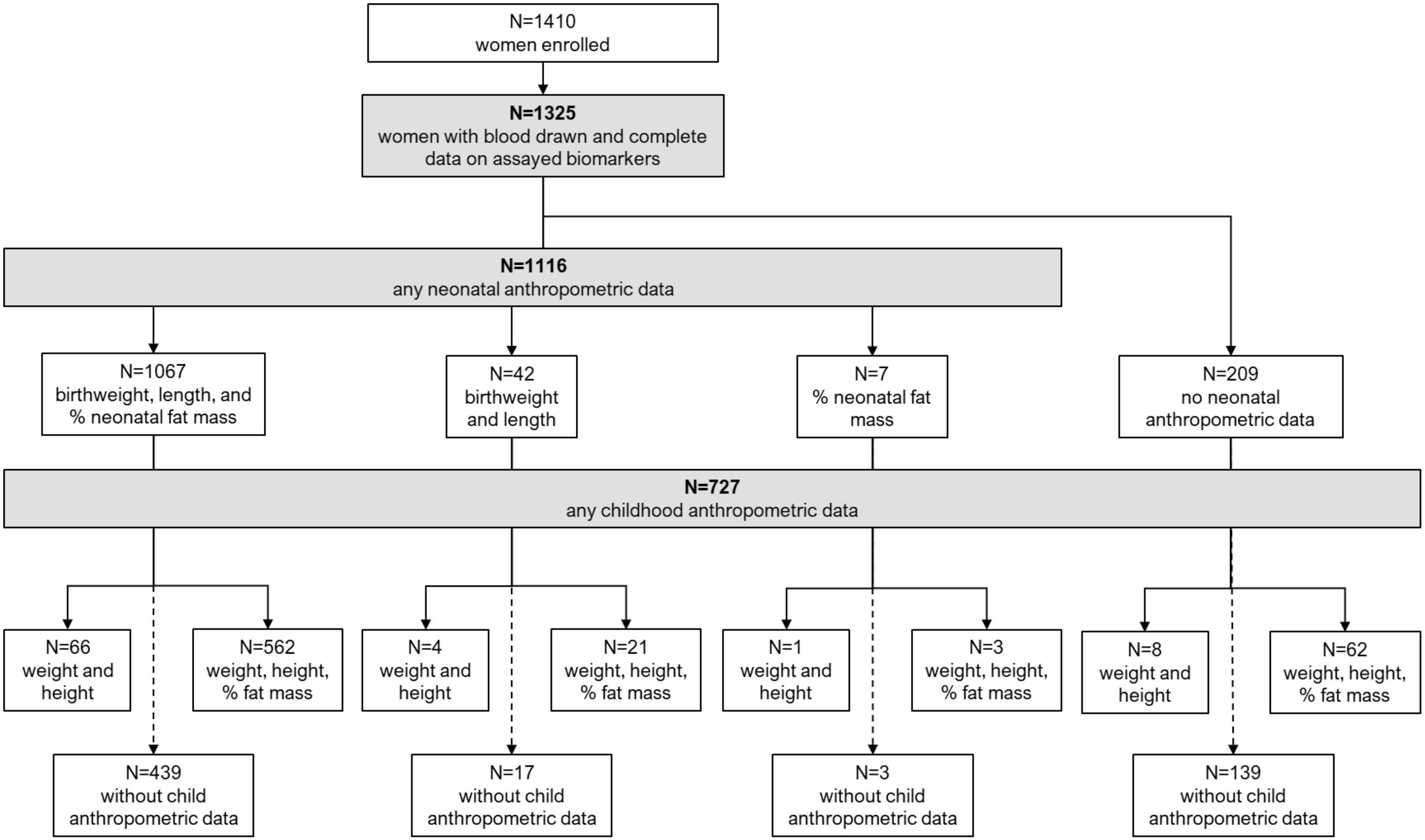

© 2023 Francis EC et al. *JAMA Network Open.*
