# Supplementary material for: Novel Metabolic Subtypes in Pregnant Women and Risk of Early Childhood Obesity in Offspring
Source: JAMA Netw Open. 2023 Apr 4;6(4):e237030. doi: 10.1001/jamanetworkopen.2023.7030 (PMC10074224; doi:10.1001/jamanetworkopen.2023.7030)
Supplement: Supplement 2. — Data Sharing Statement [file jamanetwopen-e237030-s002.pdf]

Data Sharing Statement

Francis. Novel Metabolic Subtypes in Pregnant Women and Risk of Early Childhood Obesity in Offspring. *JAMA Netw Open*. Published April 04, 2023.
doi:10.1001/jamanetworkopen.2023.7030

Data
Data available: Yes
Data types: Deidentified participant data
How to access data: anna.bellatorre@cuanschutz.edu
When available: With publication

Supporting Documents
Document types: None

Additional Information
Who can access the data: Scientific researchers
Types of analyses: For scientific research
Mechanisms of data availability: Upon reasonable request
